# Effect of overexpression of β- and γ-actin isoforms on actin cytoskeleton organization and migration of human colon cancer cells

**DOI:** 10.1007/s00418-014-1199-9

**Published:** 2014-02-28

**Authors:** Aleksandra Simiczyjew, Antonina Joanna Mazur, Agnieszka Popow-Woźniak, Maria Malicka-Błaszkiewicz, Dorota Nowak

**Affiliations:** Department of Cell Pathology, Faculty of Biotechnology, University of Wrocław, Joliot-Curie 14a, 50-383 Wrocław, Poland

**Keywords:** Actin isoforms, Cancer cells invasion, Migration

## Abstract

Actins are eukaryotic proteins, which are involved in diverse cellular functions including muscle contraction, cell motility, adhesion and maintenance of cell shape. Cytoplasmic actin isoforms β and γ are ubiquitously expressed and essential for cell functioning. However, their unique contributions are not very well understood. The aim of this study was to determine the effect of β- and γ-actin overexpression on the migration capacity and actin cytoskeleton organization of human colon adenocarcinoma BE cells. In cells overexpressing β- or γ-actin, distinct cytoskeletal actin rearrangements were observed under the laser scanning confocal microscope. Overexpressed actins localized at the submembranous region of the cell body, especially near to the leading edge and on the tips of pseudopodia. The cells transfected with plasmids containing cDNA for β- or γ-actin were characterized by increased migration and invasion capacities. However, the migration velocity was statistically significantly higher only in the case of γ-actin overexpressing cells. In conclusion, the increased level of β- or γ-actin leads to actin cytoskeletal remodeling followed by an increase in migration and invasion capacities of human colon BE cells. These data suggest that expression of both actin isoforms has an impact on cancer cell motility, with the subtle predominance of γ-actin, and may influence invasiveness of human colon cancer.

## Introduction

The actin family consists of highly conservative proteins, abundant in all eukaryotic cells. Actin plays different roles in cell functioning including cell motility, contractile ring formation during cytokinesis, maintenance of cell shape, signal transduction, cell adhesion, transcription and muscle contraction (Perrin and Ervasti 2010). In vertebrates, six actin isoforms are expressed. They have been classified by both isoelectric point and primary tissue or cellular localization and comprise β- and γ-cytoplasmic, α-skeletal, α-cardiac, and α- and γ-smooth muscle isoactins (Vandekerckhove and Weber 1978). Muscle actins are tissue specific, whereas β- and γ-cytoplasmic actins are ubiquitously present in almost all cell types and are essential for cell survival (Harborth et al. 2001). Actin isoforms are products of separate genes, although there is a high homology among their nucleotide sequences resulting in very similar protein primary structure. Actin isoforms mirror tissue but not species specificity (Sheterline et al. 1995; Khaitlina 2001). The differences between actin isoforms occur especially in the most variable N-terminal region of the actin molecule. The two cytoplasmic actin isoforms—β and γ—differ only by four amino acids located at positions 2, 3, 4 and 10. β-actin contains Asp-Asp-Asp at the N-terminus and Val at position 10 of the polypeptide chain, whereas γ-actin possesses N-terminal tripeptide Glu-Glu-Glu and Ile at position 10 (Vandekerckhove and Weber 1978). The proportion of cytoplasmic actins varies and depends on the cell type (Vandekerckhove and Weber 1978; Sheterline et al. 1995; Nowak and Malicka-Błaszkiewicz 1999). In most cells, regardless of their different origins, β- and γ-actin isoforms are found in a ratio of approximately 2:1 (Khaitlina 2007; Bergeron et al. 2010). However, the β/γ isoform ratio in different rat tissues is: 1:1 in testicles, 2.5:1 in liver and 6:1 in aorta (Vandekerckhove and Weber 1978; Nowak and Malicka-Błaszkiewicz 1999). Mammalian erythrocytes contain only β-actin (Pinder and Gratzer 1983), whereas γ-actin predominates in chicken auditory hair cells (Höfer et al. 1997). The changes in actin isoform levels in cells are often connected with pathological processes. The changed levels of β- and γ-actins were shown to accompany many tumor types such as chemically induced melanoma, hepatoma, lymphoma and breast cancer (Nowak and Malicka-Błaszkiewicz 1999; Toh et al. 1977; Gabbiani et al. 1984; Brittingham et al. 1997). Cytoplasmic β-actin seems to be overexpressed in many tumors, especially in actively moving cancer cells. This isoform level was also remarkably increased in the case of selected, highly invasive variants of colon cancer cells (Nowak et al. 2005), MDCK cells transformed by Moloney sarcoma virus (MSV) (Le et al. 1998) or melanoma T1C1 cells (Goidin et al. 2001). However, the published data are often controversial. For example, lymphocytes synthesize β-actin in remarkable excess to γ-actin, while their leukemic counterparts contain both isoforms in equal proportions. In contrast, M1 myeloblastic leukemia cells contain mainly β-actin, and only after the induction of differentiation is a higher expression level of the γ-actin gene observed in these cells (Nagata and Ichikawa 1984). Additionally, it was shown that during epithelial-mesenchymal transition in cervical carcinoma cells, reorganization of β-actin structures and downregulation of this isoform expression occur (Shagieva et al. 2012). The level of different actin isoforms’ expression can be up- or down-regulated not only in neoplastic transformation and other cell pathologies, but also in normal physiological processes observed during embryogenesis and aging (Nowak and Malicka-Błaszkiewicz 1999; Khaitlina 2001).

The existence of several actin isoforms within tissues and even within a single cell suggests functional differences among them. Cytoplasmic actin isoforms play unique roles in many processes, i.e., regulation of structure and permeability of epithelial junctions (Baranwal et al. 2012) or meiosis of oocytes (Brockmann et al. 2011). There is also some evidence that β- and γ-actins are located in different cytoplasmic areas (Khaitlina 2001). Additionally, distinct localization of β- and γ-actin mRNA in several cell types implies distinct spatial regulation of these isoforms (Hill and Gunning 1993; Watanabe et al. 1998). Cytoplasmic β-actin was identified accumulating in the region of moving cytoplasm and appeared at the tips and edges of protrusive structures responsible for active cell movement, lamella and pseudopod formation, and wound healing. This isoform also seems to play an essential role in the “ameboid” type of movement, characteristic for intravasation of cancer cells through the vessel wall (Khaitlina 2001; Le et al. 1998; Peckham et al. 2001). γ-Actin is more likely to be found in the stress fibers responsible for maintenance of cell shape, differentiation and mechanical resistance (Nowak and Malicka-Błaszkiewicz 1999; Khaitlina 2001; Otey et al. 1986). In a conflicting report, Dugina et al. (2009), using a different fixation technique and newly generated antibodies directed against cytoplasmic actin isoforms, showed that β-actin predominates in stress fibers and at cell–cell contacts between normal cells and fibroblasts, while γ-actin is present at the leading edge. The authors suggest that β-actin is responsible for cell attachment and contraction, whereas γ-actin is connected with cell motility (Dugina et al. 2009). Because of these controversies, the role of β- and γ-actins is still being examined. There are studies describing both actin isoforms’ silencing and overexpression (Peckham et al. 2001; Schevzov et al. 1992; Shmerling et al. 2005; Belyantseva et al. 2009; Bunnell and Ervasti 2010), but they did not give a clear answer to the question of their functional diversification. In cited publications, either only one isoform was knocked down (Belyantseva et al. 2009; Bunnell and Ervasti 2010) or overexpressed (Peckham et al. 2001), or the studies focused on normal cells (Dugina et al. 2009; Schevzov et al. 1992). Because of that we decided to trigger overexpression of the β- or γ-actin isoform in the human colon cancer cell line BE, representing the mesenchymal mode of motility, and to observe its effects on cell migration and invasion capacities.

## Materials and methods

### Materials

Mouse, monoclonal anti-β (clone AC-15) and anti-γ (clone 2–2.1.14.17) actin antibodies corresponding to epitopes located in the N-terminal end of β- or γ-actin, respectively, and mouse, monoclonal anti-β-tubulin antibodies were purchased from Sigma-Aldrich. Mouse, monoclonal anti-GFP antibody and sodium butyrate were obtained from Santa Cruz Biotechnology. DNase I conjugated with Alexa Fluor^®^ 594 used to detect unpolymerized actin and Alexa Fluor^®^ 568-conjugated phalloidin were obtained from Invitrogen. Secondary anti-mouse antibodies conjugated with DyLight^®^ 549 were purchased from Jackson ImmunoResearch, and anti-mouse HRP-linked antibodies were from Cell Signaling. Fetal bovine serum (FBS), trypsin, glutamine, penicillin/streptomycin, G-418 (Geneticin), DMEM and alpha-MEM media and Lipofectamine™ 2000 were purchased from Invitrogen. DNA from calf thymus and DNase I from bovine pancreas were from Sigma-Aldrich. Dako cytomatic fluorescent mounting medium was obtained from Dako. Matrigel™ and Epidermal Growth Factor (EGF) were obtained from BD Biosciences. All other chemicals were classified as analytical grade reagents.

### Cell culture

The human colon adenocarcinoma cell line BE was a kind gift of Prof. E. Sahai. The cells were grown in DMEM medium containing 10 % FBS, 2 mM glutamine and antibiotics (100 U/ml penicillin, 100 μg/ml streptomycin). Cells were cultured in 25 cm^2^ tissue culture flasks (Sarstedt) at 37 °C in 5 % CO_2_/95 % humidified air and passaged twice a week using 0.25 % trypsin/0.05 % EDTA solution.

### pAcGFP-β-actin and pAcGFP-γ-actin constructs and transfection procedure

pAcGFP-C1 β- and γ-actin constructs were generated by cloning the cDNA encoding cytoplasmic human β- or γ-actin, respectively, with their 3′UTRs (untranslated regions), into the pAcGFP-C1 plasmid (Clontech), leading to constructs whose expression resulted in fusion proteins in which the AcGFP moiety is at the N-terminal end of the actin polypeptide chain. The cDNA was amplified from the expression pOTB7 vector encoding β- or γ-actin (ATCC^®^) by using the following primers for insertion at the BamH I restriction site into the pAcGFP-C1 plasmid: β-actin forward: 5′-CATGGATGATGATATCGCCGCG-3′ and reverse: 5′-CCTAAGGTGTGCACTTTTATTCAAC-3′; γ-actin forward: 5′-CATGGAAGAAGAGATCGCCGC-3′ and reverse: 5′-CGGTTACGGCAGCACTTTTATTTT-3′. The correctness of DNA constructs was verified by sequencing. Lipofectamine™ 2000, a liposomal transfection reagent, was used to transfect the BE cells with plasmid pAcGFP-C1 encoding human β- or γ-actin and the empty pAcGFP-C1 plasmid according to the manufacturer’s protocol. 24 h after transfection cells were used for further experiments. Cells transfected with the empty pAcGFP-C1 plasmid and thus expressing AcGFP constitute control cells. Stable clones were generated by selection in complete DMEM medium containing Geneticin (G418, 1 mg/ml) according to the manufacturer’s protocol. Stable clones were incubated with 1.75 mM sodium butyrate for 72 h in order to enhance CMV (cytomegalovirus) promoter activity. Expression of cytoplasmic actin isoforms in all transfected cells was monitored by real-time PCR and Western blotting methods. For further experiments, representative clones of the cells overexpressing cytoplasmic actins were chosen.

### qRT-PCR analysis

Total RNA was extracted using NucleoSpin^®^ RNA II Kit (Macherey–Nagel) and reverse transcription reaction was performed using 0.5 μg of RNA and the High Capacity cDNA Reverse Transcription Kit (Applied Biosystems) following the manufacturer’s instructions. Negative controls and primer specificity controls were done by conducting PCR and analysis of PCR products in 1 % (w/v) agarose gel in Tris–acetate-EDTA (TAE) buffer. 5 μl of 20 times diluted transcribed cDNA was used for the subsequent qRT-PCR (quantitative reverse transcription polymerase chain reaction) with primers listed in Table 1; the reaction final volume was 15 μl. Real Time 2xHS-PCR Master Mix SYBR^®^ A (A&A Biotechnology) was used for qRT-PCR reactions, which were carried out in a Roche LightCycler^®^ 2.0 (Roche) under the following conditions: initial denaturation 3 min 95 °C, 45 cycles of denaturation (10 s at 95 °C), annealing (10 s at Tm) and elongation (22 s at 72 °C); melting curve (15 s at 65–95 °C). Error and efficiency values for each mRNA standard curve were <0.009 and >1.8, respectively, except for *ACTG*1, where the efficiency was 1.789. For quantification, the samples were normalized against the expression of *GAPDH*, *TUBA1C*, *HSP90AA1* and *18S* mRNA. It was done since, e.g., *GAPDH* was not stably expressed in BE cells. Primer sequences and their Tms (melting temperatures) are listed in Table 1. In order to estimate content of exogenous actin mRNA in total amount of actin mRNA, first we obtained standard curves with usage of all four primers pairs, where as templates served plasmids coding for either β- or γ-actin. Next, cDNAs of transfected cells were 100× diluted and subjected to qRT-PCR. Finally, results were analyzed referring to new standard curves by comparison of values obtained for AcGFP-actin mRNA to those obtained for total actin mRNA. Concentration ratio represented content (%) of exogenous actin mRNA in total actin mRNA. All experiments were done in triplicate.

**Table 1:** Nucleotide sequences, amplicon sizes and annealing temperatures (Tm) of used primers in qRT-PCR analysis

Primer	Sequence	Amplicon size (nt)	Tm (°C)
*ACTB*_f	5′ tttcttgacaaaacctaacttgcgc 3′	174	61
*ACTB*_r	5′ attgtgaactttgggggatgctc 3′		
*ACTG*1_f	5′ cagaaggagatcaccgccctg 3′	222	64
*ACTG*1_r	5′ atgcagcaaatgctacgcatctg 3′		
AcGFP-ACTB_f	5′ ctgcccgataaccactacctg 3′	222	57
AcGFP-ACTB_r	5′ gagcgcggcgatatcatcatc 3′		
AcGFP-ACTG1_f	5′ ctgcccgataaccactacctg 3′	222	57
AcGFP-ACTG1_r	5′ cagcgcggcgatctcttcttc 3′		
*GAPDH*_f	5′ ggtcgtattgggcgcctggtc 3′	252	64
*GAPDH*_r	5′ gacgtactcagcgccagcatcg 3′		
*HSP90AA1*_f	5′ gcccagttgatgtcattgatcat 3′	199	61
*HSP90AA1*_r	5′ ccacaatagtgagagttcgatcttg 3′		
*TUBA1C*_f	5′ gcagaccccttcaagttctagtca 3′	95	64
*TUBA1C*_r	5′ gtagagctcccagcaggcatt 3′		
*18S*_f	5′ gaagggcaccaccaggagtg 3′	209	64
*18S*_r	5′ gtcgcgtaactagttagcatgcc 3′		

### Isolation of cytosolic fractions

Cells were homogenized and the cytosolic fraction was prepared as described earlier by Malicka-Błaszkiewicz and Roth (1981). The cells transiently overexpressing AcGFP, AcGFP-β- or γ-actin, grown in tissue culture dishes, were gently washed with PBS (phosphate buffered saline), scraped with a rubber policeman and suspended in freshly made monomeric actin stabilizing buffer, containing 10 mM Tris–HCl, pH 7.4; 1 mM dithiothreitol; 0.1 mM ATP; 0.1 mM CaCl_2_; and 0.25 M sucrose (buffer A). Cells were centrifuged (1,000×*g*, 3 min, 4 °C) and homogenized with 3 volumes of freshly made buffer A with a Dounce homogenizer. Homogenates were centrifuged at 105,000×*g* for 1 h at 4 °C. High-speed supernatant was used as the cytosolic fraction and stored at −70 °C for further experiments. All experiments were done in triplicate.

### Isolation of cellular extracts

For Western blotting analysis, the BE cells were lysed with cytoskeletal-bound protein extraction buffer (10 mM Tris–HCl, pH 7.4, 100 mM NaCl, 1 mM EDTA, 1 mM EGTA, 1 mM NaF, 20 mM Na_4_P_2_O_7_, 2 mM Na_3_VO_4_, 1 % Triton X-100, 10 % glycerol, 0.1 % SDS, 0.5 % sodium deoxycholate) on ice. Then, cells were threefold frozen-thawed and centrifuged at 10,000×*g* for 10 min at 4 °C; supernatants were stored at −70 °C.

### Western blot analysis

Protein concentration in cellular extracts was determined by the standard Bradford procedure (Bradford 1976). Samples of an identical amount of protein (30 μg) were separated by 12.5 % polyacrylamide gel electrophoresis in the presence of sodium dodecylsulfate (SDS-PAGE) according to Laemmli (1970), followed by transfer to nitrocellulose membrane, using the procedure described by Towbin et al. (1979). Monoclonal mouse anti-GFP antibodies were used for AcGFP-actin (70 kDa) and AcGFP (27 kDa) identification. Monoclonal mouse anti-β-actin antibodies and monoclonal mouse anti-γ-actin antibodies were used for endogenous β-actin (43 kDa) and endogenous γ-actin (43 kDa) identification. The β-tubulin, recognized by monoclonal mouse anti-β-tubulin antibodies, was used as an internal loading control. Goat anti-mouse antibodies conjugated to horseradish peroxidase (HRP) were applied according to the manufacturer’s protocols. Immunoblots were developed using the Western blotting Luminol Reagent (Santa Cruz Biotechnology). Then, blots were scanned (ChemiDoc, Bio-Rad). All experiments were done in triplicate.

### Confocal microscopy

The subcellular distribution of actin filaments, β- and γ-actins and DNase I (a marker of monomeric actin) in cancer cells was examined by fluorescence staining and using a confocal laser scanning microscope (Olympus FV 500). The cells were seeded on sterile coverslips in 24-well plates and grown for 24 h. Next, the cells were transfected and 24 h later fixed with 4 % formaldehyde for 20 min at room temperature and permeabilized with 0.1 % Triton X-100 in PBS or with methanol in the case of staining with antibodies recognizing β- or γ-actin for 5 min. After fixation, coverslips were blocked for 30 min with 1 % bovine serum albumin in PBS. Monoclonal anti-β-actin and anti-γ-actin antibodies, followed by DyLight^®^ 549-conjugated anti-mouse secondary antibodies, were applied to visualize cytoplasmic actins. Actin filaments were stained with Alexa Fluor^®^ 568-labeled phalloidin, and monomeric actin was visualized with DNase I conjugated with Alexa Fluor^®^ 594. After incubation and washing steps, coverslips were mounted with Dako cytomatic fluorescent mounting medium. The overexpression of β-actin and γ-actin was observed by confocal microscopy as fluorescence of the fusion protein (AcGFP-β-actin and AcGFP-γ-actin). In each case, about 25 cells were photographed every time in three independent experiments and representative cells are shown.

Additionally, quantitative analysis of areas of transfected cells was performed, where accumulation of exogenous actins and filamentous actin was observed. We performed the analysis with the help of ImageJ software. In each case, 20 cells were analyzed.

### Evaluation of actin polymerization state

The actin content was determined by the inhibition of DNase I from bovine pancreas under standard assay conditions (Malicka-Błaszkiewicz and Roth 1981). The concentration of monomeric (G) actin was estimated by DNase I inhibition, directly in the cytosolic fraction of the cells. Total (T) actin content was measured after dilution of the samples with G actin stabilizing buffer (buffer A). For the measurement of maximal inhibition, a specific dilution below the critical actin concentration had to be applied to completely depolymerize the filamentous (F) actin. The amount of F actin was calculated by subtracting the amount of G actin from the total actin (F = T − G). The state of actin polymerization was defined by the F actin to G actin ratio (F:G). One unit of DNase I inhibitor (actin) is the amount that reduces the activity of 20 ng of DNase I by 10 % under standard assay conditions (Malicka-Błaszkiewicz and Roth 1981; Malicka-Błaszkiewicz 1986). Actin concentration was expressed in units of DNase I inhibitor per mg of sample protein. The experiments were performed three times, each as an independent experiment. Each independent experiment consisted of three measurements/probes.

### Migration assay

Cell migration tests were performed using Transwell™ filters (BD Biosciences) in a 24-well plate. For migration, control cells (transfected with pAcGFP-C1 plasmid) and cells transiently overexpressing β- or γ-actin were starved for 6 h in serum-free DMEM medium. The lower compartment was filled with 500 μl of medium consisting of 80 % DMEM, 20 % FBS and 5 nM EGF. Prior to assay cells (5 × 10^4^) were seeded onto a rehydrated Transwell™ insert in medium without FBS and EGF. After 24 h, non-migrating cells on the upper side of the filters were removed. Cells that migrated through the membrane were fixed with 4 % formaldehyde, stained with Hoechst 33342 (Invitrogen) and counted under a fluorescent microscope. The results are presented as a relative migration factor (%), where control cells that migrated through the Transwell™ filters are presented as 100 %. The experiments were performed three times. Each independent experiment consisted of three measurements.

### Invasion assay

Cell invasion tests were performed using the Boyden chamber assay. Transfected BE cells (5 × 10^4^) after 6 h of starvation in serum-free DMEM were seeded onto Transwell™ filters (BD Biosciences) coated with Matrigel™ (1 mg/ml in serum-free DMEM). Medium containing 20 % of FBS and 5 nM EGF was used in the lower compartment as a chemoattractant. After 24 h, non-migrating cells on the upper side of the filters were removed together with Matrigel™. Cells that migrated through the membrane were fixed with 4 % formaldehyde, stained with Hoechst 33342 (Invitrogen) and counted under a fluorescent microscope. The results are presented as the relative migration factor (%), where control cells that migrated through the Transwell™ filters are presented as 100 %.

### Wound healing assay

BE control cells and cells overexpressing β- or γ-actin were grown to confluence in 24-well plates. Then, the cells were wounded across the cell monolayer by scraping away a swathe of the cells with a pipette tip. Afterward, the cells were rinsed twice with PBS to remove any wound-derived, loose and dislodged cells and further cultured in a fresh medium containing 2 % FBS for 24 h. Cell migration analysis was performed using an inverted microscope (Axiovert 200 M, Zeiss) equipped with a transparent environmental chamber (Climabox, Zeiss) under 5 % (v/v) CO_2_ in air at 37 °C. The microscope was driven by the Metamorph software (Roper Scientific), and images were recorded with a CCD camera (Coolsnap HQ, Roper Scientific) every 30 min for 24 h. Cell migration was characterized and quantified using an interactive tracking method as described by Zahm et al. (1997).

### Statistical analysis

All data are given as means ± standard deviations (SD), and their significance was determined with two-tailed, unpaired Student’s *t* test. The significance level was set at *P* < 0.05.

## Results

### Transfection of BE colon cancer cells with pAcGFP-C1-β-actin and pAcGFP-C1-γ-actin plasmids

**Fig. 1:**
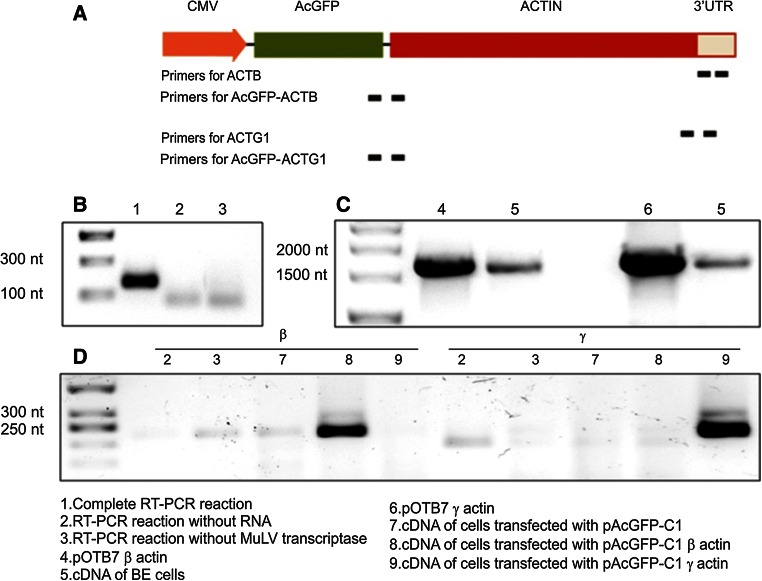
Molecular biological strategy for qRT-PCR analysis. **a** A schematic map of a part of pAcGFP-C1-actin vector, CMV—cytomegalovirus promoter. Sites to which specific primers anneal are shown as *bold bars*. **b** Control PCR reactions after RT-PCR on mRNA isolated from BE cells proving there were no contaminations while performing qRT-PCR analysis. Primers recognizing cDNA of β-actin were used, expected band size: 174 nt. **c** Control PCR reactions where pOTB7 plasmids with clones of β- and γ-actin and cDNA of BE cells served as templates; for this reaction, primers for subcloning of isoactins into pAcGFP-C1 were used. Expected band size 1,724 nt. **d** Control PCR reactions, where cDNAs of control and transfected cells served as templates and those recognizing cDNA of AcGFP-actins were taken as primers (β for AcGFP-β-actin, γ for AcGFP-γ-actin). Expected band sizes: 222 nt

**Fig. 2:**
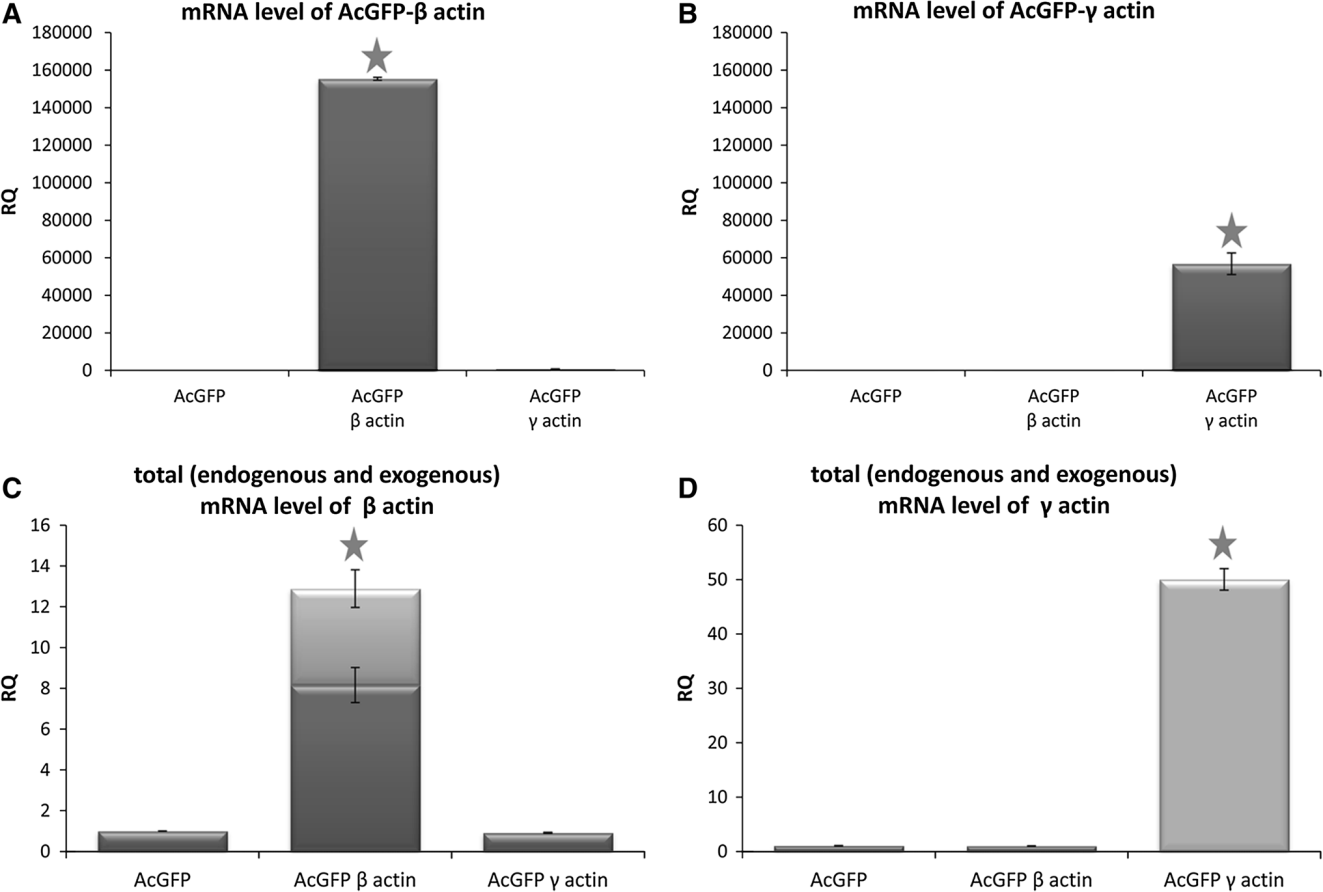
mRNA levels of actin isoforms in cells transiently transfected with plasmids coding for β- or γ-actin. The mRNA levels of AcGFP-β-actin (**a**) or AcGFP-γ-actin (**b**) and total β- (**c**) or γ-actin (**d**) were measured by qRT-PCR. On pictures **c** and **d**, we showed content of endogenous (*dark gray*) and exogenous (*light gray*) mRNA of actin in total actin mRNA level. *Asterisks* indicate values statistically different from those obtained for the control cells, transfected with pAcGFP-C1 plasmid. The significance level was set at *P* ≤ 0.05 in Student’s *t* test

**Fig. 3:**
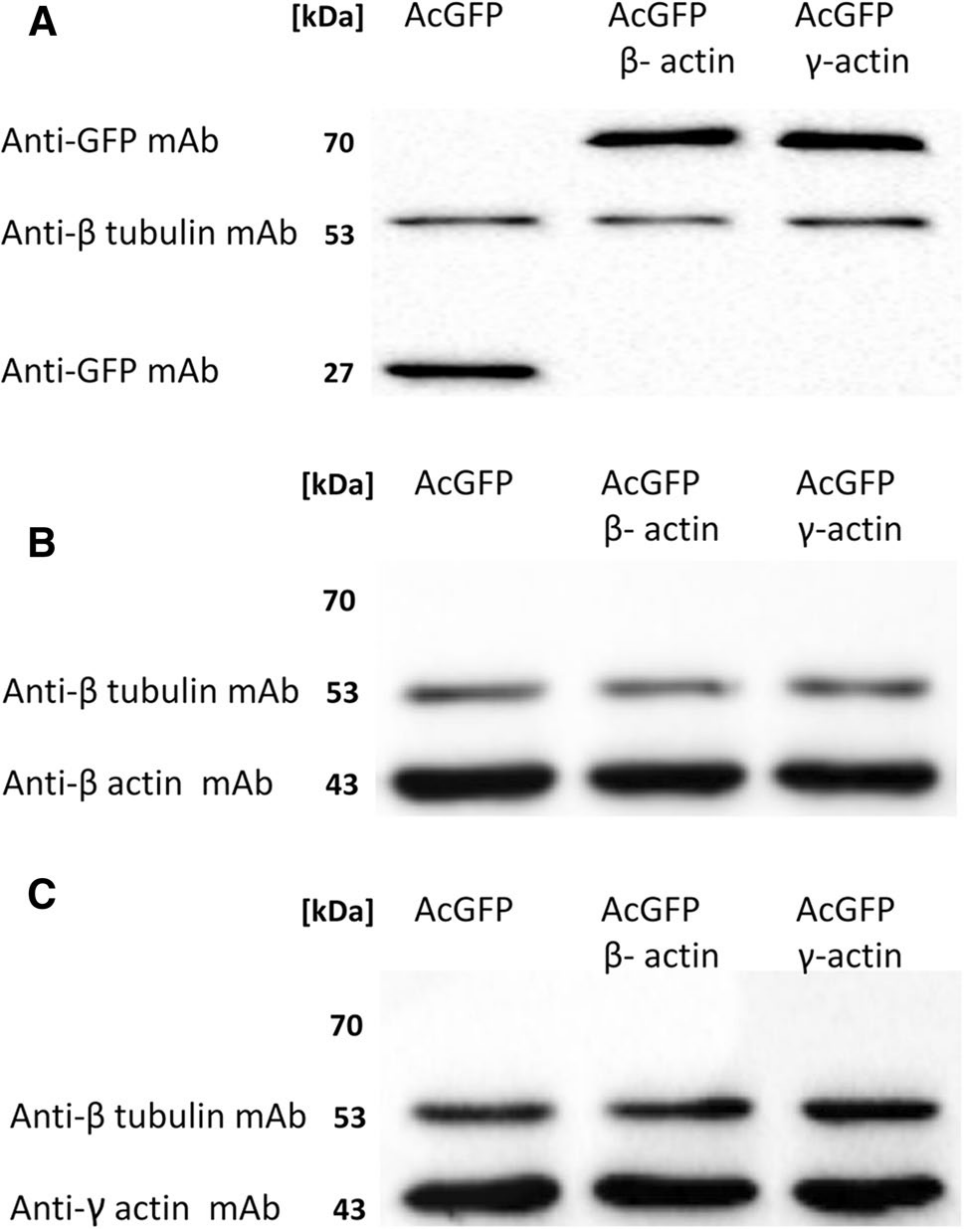
Western blot analysis of AcGFP, β-actin and γ-actin. A representative immunoblots identificating AcGFP and fusion proteins (**a**) as well as β-actin (**b**) and γ-actin (**c**) in cellular extracts of control cells (transfected with pAcGFP-C1) and cells overexpressing AcGFP tagged β- or γ-actin. Used antibodies: mouse monoclonal antibodies directed against β tubulin, mouse monoclonal antibodies directed against GFP, mouse monoclonal anti-β-actin antibodies and mouse monoclonal anti-γ-actin antibodies

Human colon adenocarcinoma BE cells, possessing elongated morphology and migrating mesenchymally (Sahai and Marshall 2003), were chosen for overexpression experiments. Having pOTB7 vectors carrying sequences encoding cytoplasmic actins, we cloned cDNAs of β- and γ-actins with their 3′UTRs into the pAcGFP-C1 plasmid. We decided to preserve 3′UTRs since it is known that these regions of mRNA are important for proper localization of at least β-actin in the cell (Kislauskis et al. 1994; Ross et al. 1997; Condeelis and Singer 2005). Next, having pAcGFP-C1-β-actin and pAcGFP-C1-γ-actin plasmids, we generated stable clones of BE cells. Transfected cells expressed human β- or γ-actin isoforms tagged at the N-terminus of the polypeptide chain to green fluorescent protein from *Aequorea coerulescens* (AcGFP). This fluorochrome is known to be present only as a monomer in a cell and does not form any aggregates as is often observed for GFP or EGFP (Jain et al. 2001; Gurskaya et al. 2003). In order to exclude disruption of the actin folding process by tagging AcGFP to the C-terminus (Brault et al. 1999; Rommeleare et al. 2004), we had to attach green fluorescent protein to the N-terminus of actin (Fig. 1). The presence of mRNA of fusion proteins (AcGFP-actins) was analyzed by qRT-PCR. Sequences, amplicon sizes and annealing temperatures (Tm) are listed in Table 1. Recognized sequences of primers are marked in Fig. 1a. For the recognition of endogenous actins and AcGFP-actin mRNA, at least the reverse primer annealed to the 3′UTR of actin mRNA. Though amplicons do not span two adjacent exons, no artifacts were introduced, which is proven by the analytical PCR shown in Fig. 1b. It is difficult to design primers for β- and γ-actins that are specific for a given isoactin mRNA, but our attempts were successful. We checked whether in BE cells there are mRNAs for both actins with whole 3′UTRs (Fig. 1c). In order to measure the amount of mRNA of only AcGFP-actins, we designed primers where the forward one annealed to the 3′ end of cDNA coding for AcGFP and the reverse one annealed to the 5′ end of cDNA encoding the actin isoform in the region which differentiates isoactins. As shown in Fig. 1b, the primers are specific. Though in the stable clones there were mRNAs of exogenous actins, at the protein level we could not observe significant green fluorescence of expressed fusion proteins in transfected cells (data not shown). We tried to increase the amount of fusion proteins in the cells by incubating the clones with sodium butyrate, which enhances CMV promoter activity (Palermo et al. 1991; D’Aiuto et al. 2006). However, it only increased the amount of mRNA of fusion proteins (data not shown). Taking into account that the level of mRNA for β- and γ-actins (both endogenous and exogenous) was not changed in comparison to cells transfected with empty plasmid (data not shown), there was observed faint fluorescence of the cells. Western blot analysis also showed a very low signal for AcGFP-actins (data not shown). It seems that the level of β- or γ-actin mRNA is strictly controlled. Therefore, we decided to obtain BE cells transiently expressing tagged β- or γ-actin. This procedure gave a better outcome: We observed a significantly increased level of total β- or γ-actin expression in pAcGFP-β-actin or pAcGFP-γ-actin transfected cells, respectively (Fig. 2). Therefore, cells transiently overexpressing selected plasmids were chosen for further experiments. Real-time PCR (Fig. 2a–d) and Western blot analysis (Fig. 3a) were performed to confirm the overexpression of β- or γ-actin isoforms in transfected cells. These results were compared to control cells, transfected with the empty pAcGFP-C1 plasmid. Additionally, we wanted to find out how endogenous actin expression is affected by exogenous actin expression. This was determined by calculating the copy number of tagged actin mRNA and subtracting it from the total copy number for selected isoform. It showed that 24 h after transfection we could observe presence of both endogenous and exogenous β-actin mRNA, whereas γ-actin mRNA was present almost only in exogenous form (Fig. 2c, d). 37 % of total β actin mRNA came from ectopic expression of cDNA coding for β-actin, whereas almost 100 % of total mRNA for γ-actin came from expressed plasmid. Because of the fact that at the N-terminus of actins AcGFP is tagged, which excluded the use of specific antibodies directed against actin isoforms, we used monoclonal anti-GFP antibodies to detect tagged actin isoforms in cell extracts (Fig. 3a–c). Nevertheless, antibodies directed against actin isoforms let us determine endogenous actin isoforms level. It was similar in cells overexpressing β- or γ-actin in relation to control cells 24 h after transfection (Fig. 3b, c). We also noticed that the level of exogenous β-actin mRNA is twice as high in comparison to exogenous γ-actin mRNA after transfection (Fig. 2a, b). These differences are imperceptible at the protein level (see Fig. 3a). This observation could suggest that expression of cytoplasmic actin isoforms is differentially regulated.

### Effect of β- or γ-actin isoform overexpression on actin cytoskeleton organization and actin polymerization in colon BE cancer cells

**Fig. 4:**
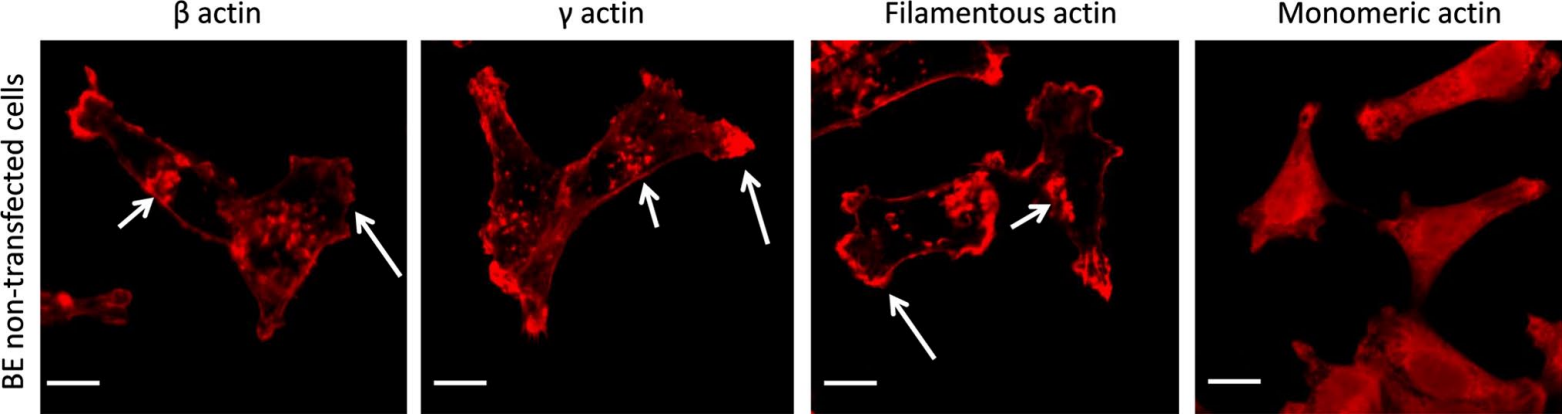
Subcellular organization of β- and γ-actins as well as filamentous and monomeric actin in non-transfected BE cells. *Left picture* β-actin stained with mouse monoclonal antibodies directed against β-actin. *Second picture* γ-actin stained with mouse monoclonal antibodies directed against γ-actin. *Third picture* filamentous actin visualized by staining with AlexaFluor^®^ 568-conjugated phalloidin. *Right picture* monomeric actin visualized by staining with DNase I conjugated with Alexa Fluor^®^ 594. *Long arrows* show localization of actin within lamellipodia and *short arrows* point at invadopodia. *Scale bar* 20 μm

**Fig. 5:**
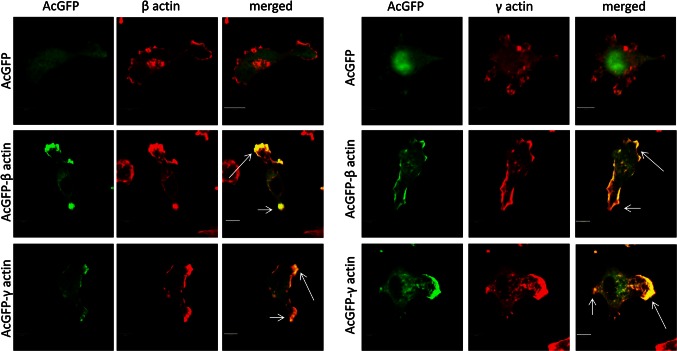
Subcellular distribution of β- (**a**) and γ-actin (**b**) in examined cells overexpressing actin isoforms. *Lower rows* in **a** and **b** shows representative BE cells overexpressing β- or γ-actin, respectively. *Left panel* AcGFP fluorescence (*green*), *middle panel* endogenous β- or γ-actin stained with mouse anti-β- or anti-γ-actin antibody (*red*). *Merged* *images* are shown in the *right* *panel*. *Long arrows* show colocalization of AcGFP-actin and endogenous actin in lamellipodia and short ones probably in retracting tail areas. *Scale bar* 10 μm

**Fig. 6:**
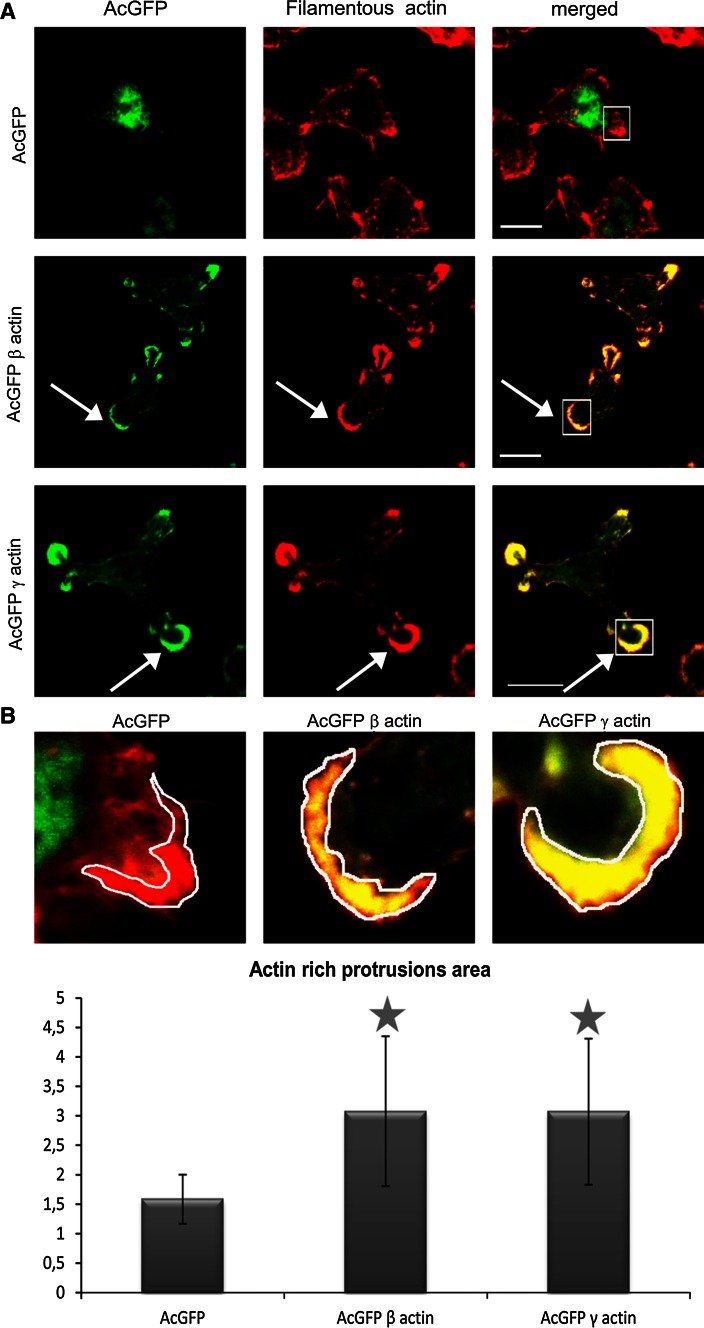
Filamentous actin organization in BE colon cancer cells overexpressing β- and γ-actin isoforms. **a** Confocal images showing cells expressing actin isoform β or γ encoded by pAcGFP-C1 expression vector were compared to cells transfected with an empty vector pAcGFP-C1. *Left panel* AcGFP fluorescence (*green*). *Middle panel* filamentous actin visualized by staining with AlexaFluor^®^ 568-conjugated phalloidin (*red*). *Merged images* are shown in the *right* panel. *Scale bar* 10 μm. *Arrows* indicate the areas of colocalization of presumed overexpressed β- or γ-actin with F actin. **b** *Upper row—*confocal images showing area of lamellipodial protrusions in all transfected cells (enlarged fragments from merged images from Fig. 5a, *rectangles squares*). *Lower row*—actin-rich protrusions areas. The data were counted for 20 cells in each case. *Asterisks* indicate values statistically different from those obtained for the control cells. The significance level was set at *P* ≤ 0.05 in Student’s *t* test

**Fig. 7:**
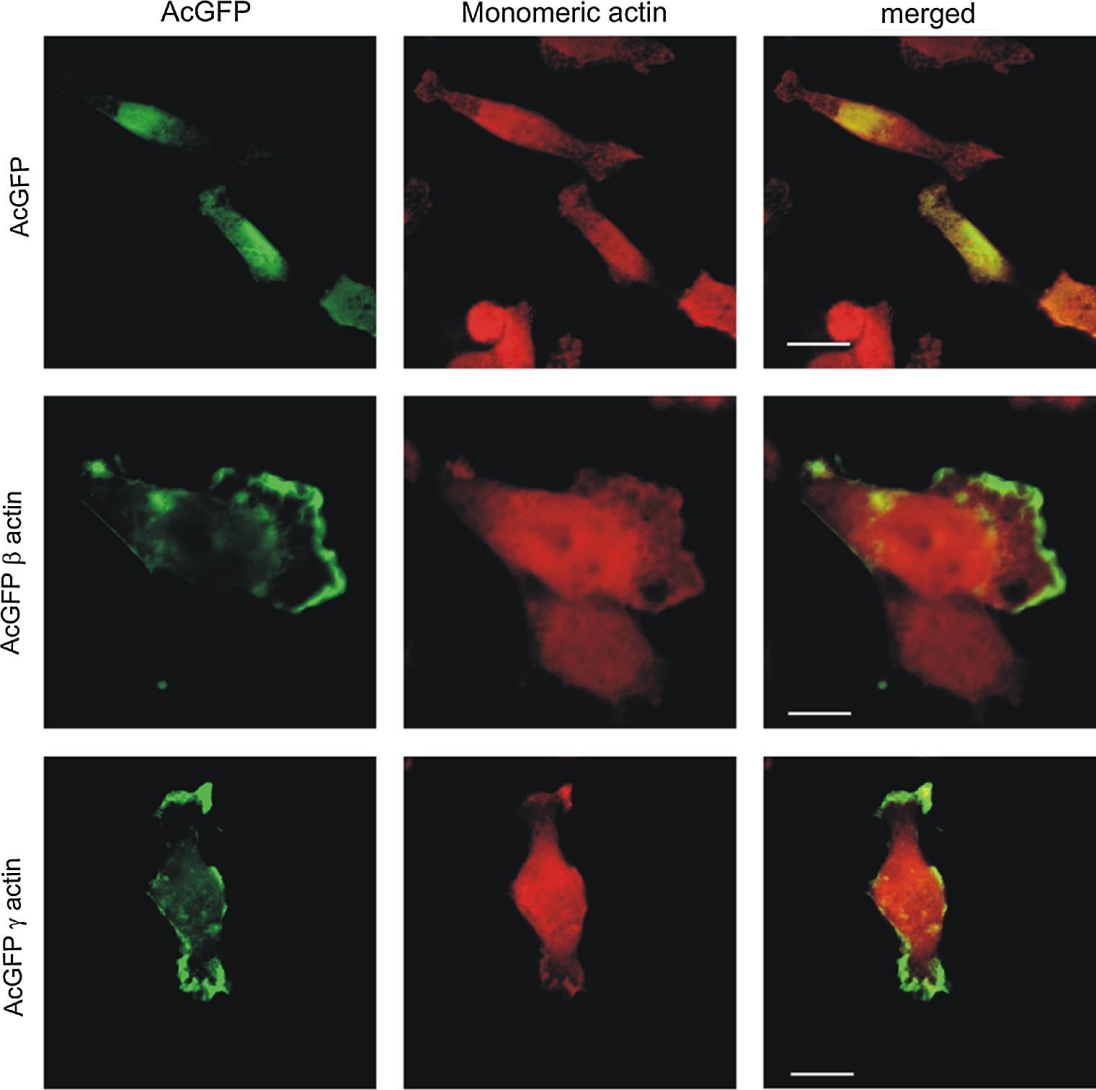
Subcellular localization of monomeric actin in cells overexpressing β- or γ-actin. β- or γ-actin was encoded by pAcGFP-C1 expression vector. *Left panel* AcGFP (*green*). *Middle panel* monomeric actin visualized by staining with DNase I conjugated with Alexa Fluor^®^ 594 (*red*). *Merged images* are shown in the *right panel*. *Scale bar* 10 μm

The cytoskeleton of non-transfected BE cells was organized in the form of a cortical ring under the cellular membrane and actin meshwork in the cell body, with clearly defined lamellipodia, invadopodia and many subtle protrusions. These cells do not form prominent stress fibers (Fig. 4). Transiently transfected BE cells were fixed, stained and analyzed by scanning confocal microscopy. All our results obtained for cells overexpressing actin isoforms were compared to control cells, transfected with an empty vector introducing only AcGFP expression. The intracellular distribution of endogenous β- or γ-actin was analyzed by staining with monoclonal antibodies recognizing β- or γ-actin, respectively. In cells transfected with the pAcGFP-β-actin or pAcGFP-γ-actin plasmid, overexpressed actin isoforms were visualized by fluorescence of green fluorescent protein (AcGFP). In both cell variants, overexpressed actins localized at the submembranous region of the cell body, especially near to the leading edge and on the tips of pseudopodia. Exogenous actins colocalized strongly with both endogenous β- and γ-actins, in contrast to AcGFP in control cells (Fig. 5, right panels, white arrows). It confirms that fusion proteins—AcGFP-conjugated actins possess proper biological activity and fulfill similar function as endogenous actin in BE cells. Our next goal was to analyze the distribution of filamentous actin (F actin) visualized by staining with phalloidin-Alexa Fluor^®^ 568 in transfected cells (Fig. 6a). The BE adenocarcinoma cells expressing pAcGFP-β-actin or pAcGFP-γ-actin were characterized by the strong colocalization of F actin and overexpressed actin isoforms, especially in the area of lamellipodia and other protrusions (Fig. 6, white arrows). The area occupied by these protrusions is statistically significantly larger in cells overexpressing β- or γ-actin than in control cells (Fig. 6b). In the next step, distribution of unpolymerized actin (G actin) was verified. Control cells and cells transfected with pAcGFP-β-actin or pAcGFP-γ-actin were stained with DNase I-Alexa Fluor^®^ 594 to analyze G actin distribution in the cells (Fig. 7). It binds much more strongly to monomeric G actin than to filamentous actin, and due to that it can be used to detect unpolymerized actin in the cell. In control cells, G actin was present in nuclei and the perinuclear area, while in cells overexpressing β- or γ-actin, it was localized additionally in the whole cell body (Fig. 7).

**Fig. 8:**
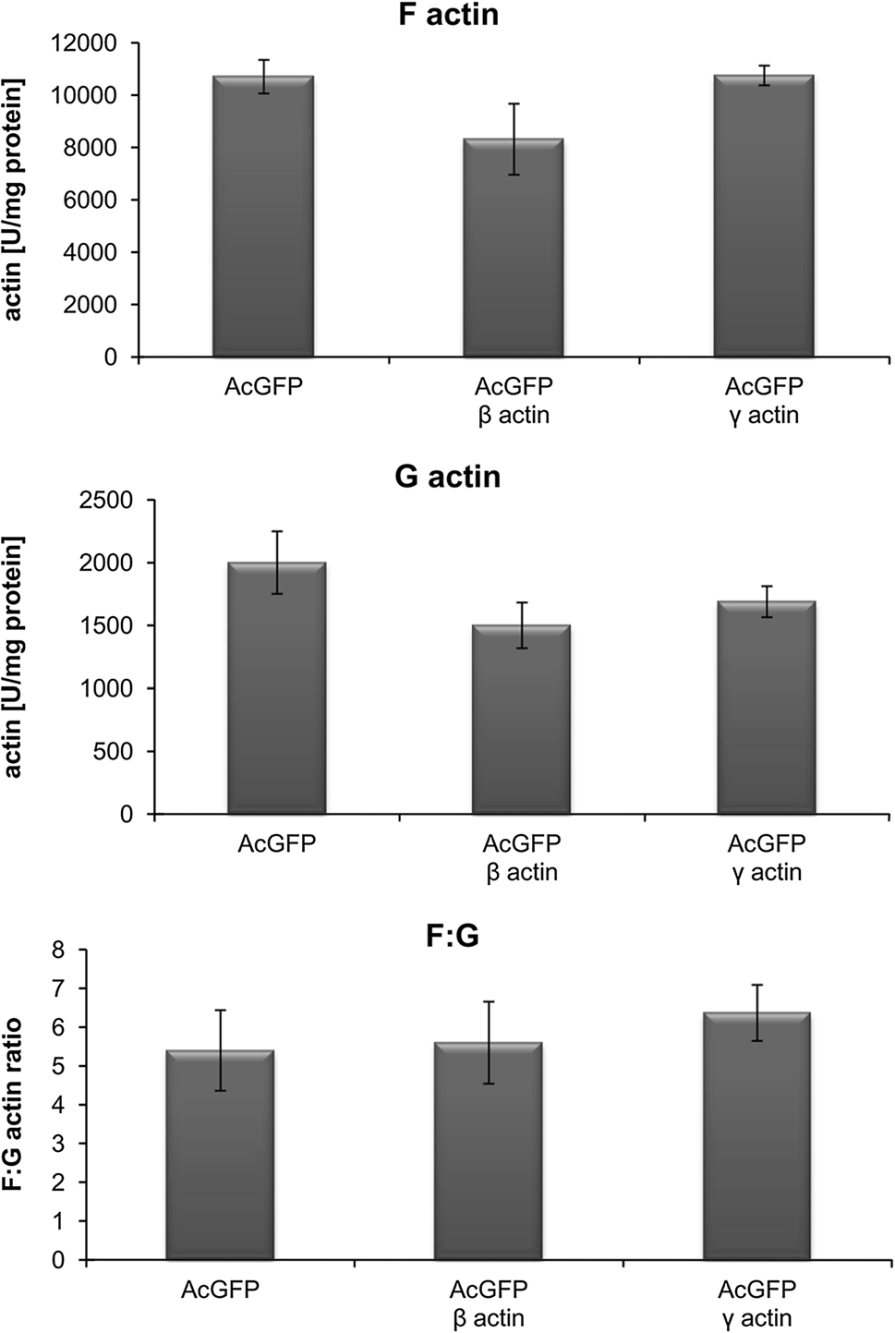
Changes in actin polymerization state in the BE cells overexpressing β- or γ-actin. Results were compared to cells transfected with an empty vector pAcGFP-C1. The data were obtained from three independent experiments

Since we did not notice a big difference between distributions of overexpressed β- and γ-actin in tested cells, we decided to check whether one of the isoforms influences the F/G ratio. Cells expressing AcGFP were used as a control. Monomeric and total actin were measured in the cytosolic fractions of examined cells by the DNase I inhibition assay under standard conditions (Malicka-Błaszkiewicz and Roth 1981). Amounts of filamentous actin and the state of actin polymerization were calculated as described in the “Materials and methods” section. The results of this analysis are shown in Fig. 8. We could observe a slight increase in the F/G actin ratio in AcGFP-γ-actin overexpressing cells, but these results are not statistically significant.

### Effects of β- and γ-actin overexpression on the migration and invasion abilities of BE cells

**Fig. 9:**
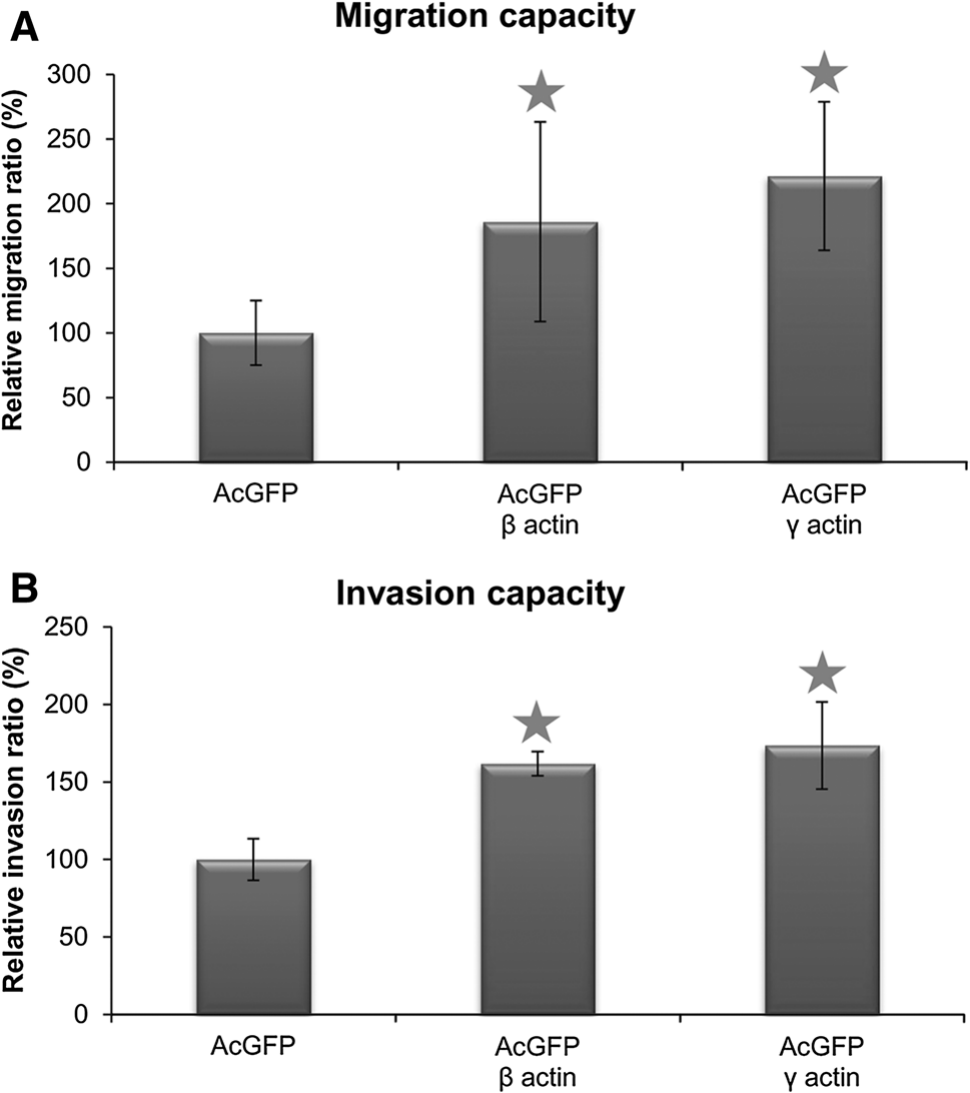
Migration (**a**) and invasion (**b**) capacities of BE cells overexpressing β- or γ-actin isoform. Results expressed as the mean ± SD are representative for at least three independent experiments. Migration and invasion in control cells are presented as 100 %. *Asterisks* indicate values statistically different from those obtained for the control, transfected with pAcGFP-C1 plasmid cells. The significance level was set at *P* ≤ 0.05 in Student’s *t* test

To investigate whether the overexpression of both actin isoforms affects the migration and invasion capacities of colon adenocarcinoma cells, a Boyden chamber migration assay was performed. The obtained results are presented in Fig. 8. A significant increase in migration capacity was observed in BE cells transfected with pAcGFP-C1-β-actin or pAcGFP-C1-γ-actin in comparison to control cells (Fig. 9a). Cells overexpressing β- or γ-actin showed a similar relative migration ratio. Invasion assay results are similar to outcomes from the migration assay (Fig. 9b).

**Fig. 10:**
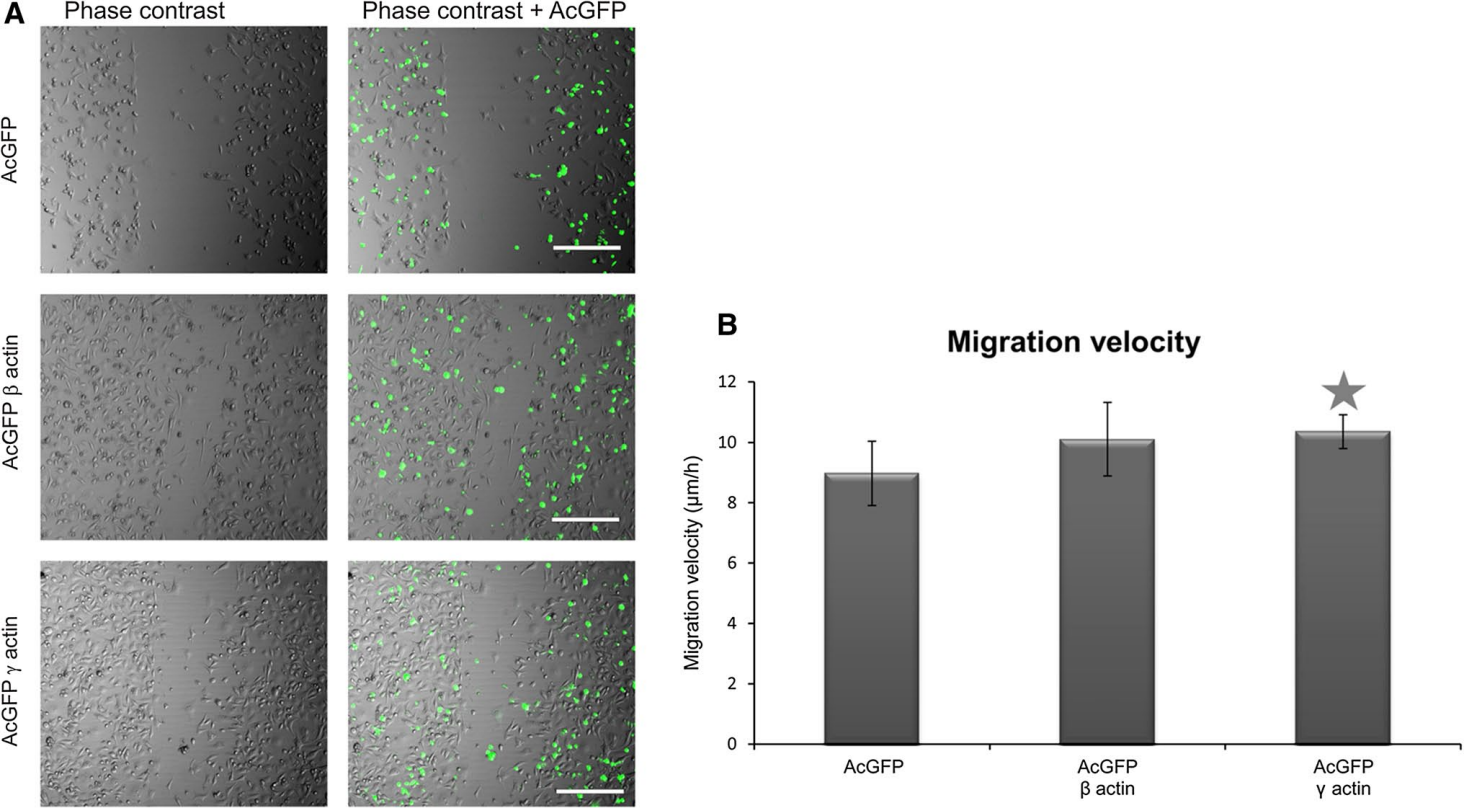
Wound healing assay of BE cells overexpressing β- or γ-actin. **a** Shows representative experiments, where photographs were taken 24 h after scratching of the cell monolayer. *Scale bar* 50 μm. Diagram **b** shows migration velocity of BE cell variants. *Asterisk* indicates value statistically different from those obtained for the control, transfected with pAcGFP-C1 plasmid cells. The significance level was set at *P* ≤ 0.05 in Student’s *t* test

To examine whether β- or γ-actin overexpression influenced the migration velocity of colon cancer BE cells, the wound healing assay was applied as described in Materials and Methods. The migration speed and process of cell migration at the wound edge during wound repair are presented in Fig. 10. The migration velocity of pAcGFP-β-actin and pAcGFP-γ-actin transfected cells demonstrated higher migration velocity than the cells overexpressing AcGFP. This difference is statistically significant only in the case of γ-actin overexpressing cells (Fig. 10b).

## Discussion

Due to the controversies concerning the unclear role of β- and γ-actin isoforms in the cell migration process, we decided to verify how overexpression of both actins would affect cytoskeletal organization and cell migration. In recent years, there have been only a few reports published describing the effects of overexpression or silencing of β- or γ-actin (Peckham et al. 2001; Dugina et al. 2009; Shum et al. 2011). We decided not to silence expression of these isoforms because of the low efficiency of this process indicated by others (Dugina et al. 2009; Shum et al. 2011). This problem could be overcome by the knockout of genes coding for actin isoforms. The effect of β- and γ-actin gene knockout was observed on fibroblasts derived from mice devoid of these isoforms (Bunnell and Ervasti 2010; Bunnell et al. 2011; Tondeleir et al. 2012). Unfortunately, it was not a good model for our purpose, because it is only possible to obtain normal cells in this way and we are interested in cancer cells lacking β- or γ-actin. Obtaining cancer cells without expression of these actin isoforms is impossible because of the fact that β-actin whole-body knockout resulted in embryonic lethality (Bunnell et al. 2011; Tondeleir et al. 2012). The γ-actin null mice were fully viable during embryonic development, but most died within 48 h after birth as a result of significant impairments (Bunnell and Ervasti 2010).

Therefore, we generated plasmids by cloning the cDNA encoding human cytoplasmic β-and γ-actins with their 3′ untranslated regions (3′UTR) into the pAcGFP-C1 plasmid. We used the pAcGFP-C1 vector instead of the pEGFP-C1 vector because unlike EGFP, AcGFP occurs always in the form of a monomer (Jain et al. 2001; Gurskaya et al. 2003). We preserved 3′UTR sequences because these regions of mRNA are important for correct localization of at least β-actin in the cell (Kislauskis et al. 1994; Ross et al. 1997; Condeelis and Singer 2005) and we wished to observe spatial segregation of actin isoforms. Due to the fact that attachment of the tag to the actin C-terminus may cause problems in its folding (Brault et al. 1999; Rommeleare et al. 2004), we chose to attach AcGFP to the N-terminus of actin preceded by linker (16-amino acid rests). After transfection, the expression of AcGFP, AcGFP-β- and AcGFP-γ-actins was observed, but the level of total β- and γ-actin isoform mRNAs in stable transfectants did not change significantly. Other researchers have obtained similar results; the efficiencies of both overexpression and silencing were also not very high in their experiments (Dugina et al. 2009; Schevzov et al. 1992; Shum et al. 2011; Choidas et al. 1998). Choidas et al. (1998) found that GFP-actin in stable transfectants constituted on average 5 % of the total actin and usually only 3–5 % of cells expressed the GFP-actin gene. In other cases, overexpression efficiency of β-actin was at the level of 60 % (Peckham et al. 2001) and the reduction in expression at the level of 31–41 % for both isoforms (Dugina et al. 2009; Shum et al. 2011). Additionally, Schevzov et al. (1992) found that β- or γ-actin transfected cells displayed a wide range of introduced cDNA expression levels. Clones expressed the exogenous cDNA in the range from 10 to 60 %. The quite low level of overexpression may be related to the fact that actin is essential for cell functioning and its level must be tightly regulated. One of the regulation mechanisms was described by Sotiropoulos et al., and it involves SRF (serum response factor) (Sotiropoulos et al. 1999; Posern and Treisman 2006). SRF is a transcription factor, a phylogenetically conserved nuclear protein, mediating the rapid transcriptional response to extracellular stimuli, e.g., growth and differentiation signals (Arsenian et al. 1998). The authors discovered that monomeric actin, which appeared in cells overexpressing actin, inhibited SRF and thereby greatly decreased expression of SRF-controlled genes, among which are also β- and γ-actin genes (Sotiropoulos et al. 1999; Posern et al. 2002). It is likely that also in the case of our cells after stable transfection with pAcGFP-β-actin or pAcGFP-γ-actin a reduction in endogenous actin expression occurred. It seems that although exogenous actin is expressed, the total actin level remains almost unchanged. Due to the fact that the level of the total β- or γ-actin in stable clones was not increased in comparison to non-transfected cells, we decided to perform transient transfection. In transiently transfected cells, the expression of total cytoplasmic actin isoforms was much higher in pAcGFP-β- or pAcGFP-γ-actin transfected cells than in control cells at both the mRNA and protein level. We found out that 24 h after transfection, in AcGFP β-actin overexpressing cells, both endogenous and exogenous β-actin mRNAs were present, whereas in AcGFP γ-actin overexpressing cells mRNA coding γ-actin was detected only for ectopically expressed cDNA encoding AcGFP γ-actin. At the same time, we could detect both endogenous actin isoforms in these cells at protein level. It can be due to the fact, that half-life of actin proteins is usually 2–3 days, so 1 day after transfection part (β-actin) or the entire (γ-actin) pool of mRNA is already in the exogenous form, while at the same time we can still observe in the cell endogenous actins as proteins (Antecol et al. 1986; Cheever et al. 2011; Dugina et al. 2009). It is not clear why the difference between the ratio of endogenous actin to exogenous actin coding β-actin and γ-actin mRNA occurred. It can be connected to distinct ways of regulation of these isoforms expression within cells. Mechanisms taking part in regulation of β actin expression, localization and function are much better known. Among them are: dependence of mRNA localization on 3′UTR (Condeelis and Singer 2005) or arginylation (Karakozova et al. 2006; Terman and Kashina 2013). Ways of controlling γ actin expression, localization and functions are still quite poorly understood. Additionally, localization and function of actin isoforms can be differentially regulated by selected actin binding proteins (De La Cruz 2005; Müller et al. 2013).

Due to the fact that spatial and temporal segregation of actin isoforms in the cytoplasm has been observed (Dugina et al. 2009; Tondeleir et al. 2012), it is likely that they can play specific and different roles in the cell. That is why we decided to verify localization of cytoplasmic actin isoforms in the examined cells. Previous reports showed that β-actin is preferentially located at the leading edge of newly formed cellular protrusions (Hoock et al. 1991; Gimona et al. 1994; Ballestrem et al. 1998), and that its mRNA is located in the lamella, proximal to the lamellipodia, where the translation process takes place (Kislauskis et al. 1994). Additionally, increased expression of β-actin and its redistribution to the tips of pseudopodia was observed in the MDCK and the LS180 invasive cell variants (Nowak et al. 2005; Le et al. 1998). This may suggest that this isoform is preferentially responsible for cell migration. γ-Actin displays a more ubiquitous distribution and could be found in the stress fibers responsible for maintenance of cell shape, differentiation and mechanical resistance (Nowak and Malicka-Błaszkiewicz 1999; Otey et al. 1986; Ulloa and Avila 1996). The comparison of actin isoforms’ localization was associated with difficulties due to the fact that they differ from each other very slightly, only by four amino acids within the N-terminus of the polypeptide chain (Vandekerckhove and Weber 1978). Because of that, obtaining specific monoclonal antibodies directed against them was for a long time problematic and difficult. Perhaps the dominant role of β-actin described in the migration process was connected with the fact that β-actin monoclonal antibodies were available (Gimona et al. 1994), whereas for a long time not enough specific antibodies directed against γ-actin were commercially sold. In recent years, this situation has changed, and some studies have focused on localization and functional diversification of both cytoplasmic actin isoforms (Dugina et al. 2009; Bunnell et al. 2011). It was shown that β-actin is present in stress fibers and is involved in cell attachment and contraction, while γ-actin can be found at the leading edge and it is connected with cell motility in normal cells (Dugina et al. 2009). Our data demonstrate that both β- and γ-actin are located in the submembranous area, which suggests that they can play an equivalent role in cell migration. Similar results were obtained by Bunnell et al. (2011) in the case of mouse embryonic fibroblasts.

The next step of our experiments was to verify whether β- or γ-actin overexpression would change their localization and function in the cell. The results of previous studies did not provide a clear answer to this question. Peckham et al. (2001) reported that myoblasts overexpressing β-actin exhibited enlarged areas of protrusion and retraction. Additionally, the level of β-actin in the newly protruded regions was raised. However, other researchers have shown that γ-actin may also be involved in cell movement. Shum et al. (2011) revealed that γ-actin knockdown in neuroblastoma cells decreased wound healing closure. Dugina et al. (2009) postulated that γ-actin is associated with cell movement in a higher degree than β-actin in normal cells. It was connected with the fact that β-actin-depleted cells highly reduced their stress fiber content, whereas γ-actin-depleted cells did not generate lamellipodial protrusions. Additionally, knockout of β-actin impaired but did not completely abolish cell migration (Tondeleir et al. 2012), which suggests that both isoforms can take part in this process. In our experiments, the BE colon cancer cells overexpressing β- or γ-actin produced broad lamellipodial membrane extensions and retracting tails in which endogenous and exogenous β- or γ-actin colocalized. This strongly indicates that both actin isoforms participate in the cellular motility of these cells and confirms that fusion proteins—AcGFP-conjugated actins possess biological activity similar to endogenous actin in BE cells.

Our studies also showed that AcGFP-β-actin and AcGFP-γ-actin colocalize strongly with filamentous actin, especially in areas related to cell migration. We did not observe similar colocalization of fusion proteins with monomeric (G) actin in transfected cells. G actin was localized mainly in the perinuclear region, where the excess actin that appeared in transfected cells as an effect of overexpression can be stored. Our data suggest that both overexpressed isoforms are present mostly in filamentous form. Observations of actin polymerization state confirmed these results. Regulation of actin polymerization plays an essential role in the process of cancer cell migration. Previous studies showed an existing correlation between the metastatic potential of human cancer cells and the state of actin polymerization (Katsatonis et al. 1994; Stournaras et al. 1996; Verschueren et al. 1994; Nowak et al. 2002; Popow-Woźniak et al. 2012; Radwanska et al. 2012). Relative migration and invasion ratios are higher for β- or γ-actin overexpressing cells in relation to control cells. The velocity of migration during wound closure is also greater for pAcGFP-β-actin and pAcGFP-γ-actin than for pAcGFP-C1 transfected cells. This difference is statistically significant only in the case of γ-actin overexpressing cells. Effects of β-actin overexpression on cell migration were analyzed by Peckham et al. (2001), who found that in myoblasts overexpression of this isoform increased cell speed to almost double that of control cells. Furthermore, Shum et al. (2011) revealed that γ-actin knockdown in neuroblastoma cells decreased migration through the Transwell™ insert and reduced migration speed. Bunnell et al. (2011) indicated β-actin as an isoform responsible for cell migration. In their research, β-actin knockout mouse embryonic fibroblasts were growth impaired and their migration velocity was significantly decreased compared with control cells. In contrast, Dugina et al. (2009) found that the average speed of cell migration was not different between control and β-actin-depleted cells, but was significantly decreased in γ-actin-depleted rat fibroblasts (~33 % decrease). This is in line with our observations of the BE colon carcinoma cells, where the overexpression of cytoplasmic γ-actin seems to have a preferential influence on migration abilities of examined cells.

It seems that overexpression of either β or γ actin has more prominent impact on cell migration through three dimensional (3D) matrix than on two dimensional (2D) surface. The amounts of cells migrating through empty or filled with Matrigel™ Transwell™ insert (3D conditions) were much higher in case of cells overexpressing actins in comparison to control cells. Whereas in the case of migration on 2D surface increase in migration velocities were relatively small while comparing cells overexpressing β- or γ-actin to control cells. A significant difference in relation to control cells was observed only for cells overexpressing γ actin. Our results indicate that the role of cytoplasmic actins in cancer cell motility could depend on complexity of cell microenvironment. This factor can influence different regulation of β- and γ-actins functions.

Migration and invasion processes in cancer cells are regulated by actin binding proteins. It is known that proteins from the ADF/cofilin family, responsible for filament severing, bind with different affinities to muscle and cytoplasmic actins (De La Cruz 2005). However, it is not known so far whether such differences exist between β- and γ-actins. Additionally, β- and γ-actin isoforms contribute to the modulation of nonmuscle myosin-2 and myosin-7A activity and thereby to the spatial and temporal regulation of cytoskeletal dynamics (Müller et al. 2013). Furthermore, actin isoforms dynamics can be regulated by the changes of pH inside the cell (dos Remedios et al. 2003). These factors could be responsible for higher velocity of γ-actin overexpressing cell migration.

In conclusion, the study of β- and γ-actins’ role in cell migration is difficult to elucidate and will last for several years. Previously, it was found that β-actin is responsible for cell movement and γ-actin plays more of a structural role and is involved in stress fiber formation, but the data were ambiguous. Our research clearly suggests that both cytoplasmic actin isoforms are involved in cell migration and invasion. The unique role of β-actin may be connected with regulation of transcription of many genes (Tondeleir et al. 2012; Zheng et al. 2009).
